# Ability of diastolic arterial pressure to better characterize the severity of septic shock when adjusted for heart rate and norepinephrine dose

**DOI:** 10.1186/s13613-025-01454-y

**Published:** 2025-03-26

**Authors:** Antoine Goury, Zoubir Djerada, Glenn Hernandez, Eduardo Kattan, Romain Griffon, Gustavo Ospina-Tascon, Jan Bakker, Jean-Louis Teboul, Olfa Hamzaoui

**Affiliations:** 1 Unité de Médecine Intensive et Réanimation Polyvalente, CHU Reims, Reims, F-51100 France; 2 Université de Reims Champagne-Ardenne, Unité HERVI “Hémostase et Remodelage Vasculaire Post- Ischémie” - EA 3801, Reims, F-51100 France; 3 Departamento de Medicina Intensiva, Facultad de Medicina, Pontificia Universidad Católica de Chile, Santiago, Chile; 4 Department of Intensive Care Medicine, Fundación Valle Del Lili, Cali, Colombia; 5 Translational Research Laboratory in Critical Care Medicine (Translab-CCM), Universidad Icesi, Cali, Colombia; 6 Department Intensive Care Adults, Erasmus MC University Hospital Rotterdam, Rotterdam, Netherlands; 7 Division of Pulmonology, Critical Care and Sleep Medicine, Columbia University Irving Medican Center, New York, USA; 8 Faculté de Médecine Paris-Saclay, Université Paris-Saclay, Le Kremlin-Bicêtre, France

**Keywords:** Septic shock, Norepinephrine, Diastolic arterial pressure, Mortality, Vascular tone, Vascular responsiveness, Heart rate

## Abstract

**Background:**

Septic shock is commonly associated with reduction in vasomotor tone, mainly due to vascular hyporesponsiveness to norepinephrine (NE). Although the diastolic arterial pressure (DAP)/heart rate (HR) ratio reflects vasomotor tone, it cannot be a reliable index of vascular responsiveness to NE (VNERi). We hypothesized that adjusting DAP/HR for the NE dose could yield a VNERi value (VNERi = DAP/(NE dose x HR)), knowledge of which can help guiding therapeutic strategies in cases of persistent hypotension despite NE (e.g., increasing NE doses vs. introducing additional vasopressors). For our hypothesis be valid, at least VNERi should demonstrate a stronger association with patient outcome than DAP, DAP/HR or mean arterial pressure (MAP)/NE dose, a global marker of NE responsiveness.

**Methods:**

We conducted a post-hoc analysis of the ANDROMEDA-SHOCK database. Hemodynamic variables and initial NE doses were recorded at the randomization time-point, within 4 h of septic shock diagnosis. NE doses were expressed in µg/kg/min (using the bitartrate NE formulation). A multivariate model was employed to compare the associations between these variables and key clinical outcomes, including in-hospital mortality, numbers of vasopressor-free days and of renal replacement therapy (RRT)-free days up to day 28.

**Results:**

The ANDROMEDA-SHOCK database included 424 patients with septic shock receiving NE. The median DAP was 52 mmHg [IQR: 45–50] and the median NE dose at inclusion was 0.2 µg/kg/min [IQR: 01-0.4]. In-hospital mortality was 43%. VNERi demonstrated the strongest association with in-hospital mortality compared to DAP, DAP/HR, and MAP/NE dose, emerging as the most significant covariate in the multivariate model. Similar findings were found for the associations with numbers of vasopressor-free days and RRT-free days up to day 28. The model revealed an inverted J-shaped relationship between in-hospital mortality and VNERi, with a nadir point at 6.7, below which mortality increased.

**Conclusions:**

In patients receiving NE during early septic shock, VNERi demonstrated the strongest association with outcome compared to DAP, DAP/HR, and MAP/NE dose. Due to its physiological basis and robust association with outcomes, VNERi may serve as a valuable bedside marker of the vascular responsiveness to NE. This index could potentially be integrated into decision-making of early septic shock.

**Supplementary Information:**

The online version contains supplementary material available at 10.1186/s13613-025-01454-y.

## Background

Sepsis-related circulatory failure results from complex mechanisms including vascular dysfunction, hypovolemia, and myocardial depression [1]. Sepsis-associated vascular dysfunction is characterized by impaired endothelial function, altered vasomotor tone, and disturbed microcirculation. Vasomotor tone dysfunction, marked by hyporesponsiveness to vasoconstrictive agents [2], results in reduced vasomotor tone (vasodilatation) and hypotension, ultimately contributing to tissue hypoperfusion [3]. Additionally, factors unrelated to vascular hyporesponsiveness to vasoconstrictors, such as the use of sedative drugs or incomplete clearance of chronically administered vasodilators, can further lower vasomotor tone at the early phase of septic shock. This underscores that vasomotor tone and vascular responsiveness to vasoconstrictors are distinct conceptual entities, meaning that markers of vasomotor tone may not reliably reflect vascular responsiveness to vasoconstrictors.

A simple bedside method to approaching vasomotor tone is to consider diastolic arterial pressure (DAP) [4]. Indeed, physiologically, peripheral vascular resistance is the primary determinant of DAP [5] with heart rate (HR) and arterial stiffness playing less significant roles [5, 6]. In septic shock, a low DAP, reflecting diminished vasomotor tone, has been proposed as a trigger to initiate administration of norepinephrine (NE) [7–10], given the α_1_-agonist effects of this agent on the vasomotor tone. Since tachycardia increases DAP due to reduced diastolic time, DAP adjusted for HR may provide a more accurate assessment of vasomotor tone than DAP alone [11]. However, neither DAP nor DAP/HR can capture vascular responsiveness to NE because: (1) both can be influenced by factors that affect vascular tone but not vascular responsiveness, as mentioned above, and (2) similar DAP/HR values may be observed in different clinical scenarios. For example, a patient (A) receiving a high NE dose (e.g., 0.5 µg/kg/min) and a patient (B) receiving a low NE dose (e.g., 0.05 µg/kg/min) might exhibit comparable DAP/HR ratios due to more marked vascular hyporesponsiveness to NE in patient (A).

In this study, we hypothesized that adjusting the DAP / HR ratio for NE dose would yield an index of vascular responsiveness to NE (VNERi = DAP / (NE dose × HR)). This index could potentially guide therapeutic strategies in cases of persistent hypotension despite NE treatment, such as helping to choose between escalating NE doses or introducing a non-catecholaminergic vasopressor or corticosteroids. For this hypothesis to be valid, at least VNERi would need to demonstrate a stronger association with clinical outcome than DAP, DAP/HR and mean arterial pressure (MAP)/NE dose, the latter being a global marker of NE responsiveness as MAP reflects both cardiac output and systemic vascular resistance (SVR).

## Materials and methods

### Study design and settings

We performed a secondary analysis of the database from the multicentre randomized ANDROMEDA-SHOCK trial [12], which has been running from March 2017 to April 2018 in 28 hospitals in Argentina, Chile, Colombia, Ecuador, and Uruguay. The respective ethical and research committee involving human beings approved the use of the randomized controlled trial [12]. Data use was subject to an agreement in accordance with French data protection law (RGPD # MR004060220233). This study report complies with the Strengthening the Reporting of Observational Studies in Epidemiology (STROBE) Statement guidelines (Supplemental Table S1).

### Patient selection

Patients included in the ANDROMEDA-SHOCK trial met al.l the criteria for septic shock defined by the Third International Consensus Definitions for Sepsis and Septic Shock [13]: any patient with suspected or proven infection, associated with life-threatening organ dysfunction, with persistent hypotension requiring vasopressors MAP > 65 mmHg, arterial lactate > 2 mmol/L despite correction of hypovolemia (at least 20 mL/kg over 60 min). Patients were recruited within 4 h of meeting these criteria. Exclusion criteria included bleeding, severe acute respiratory distress syndrome, and do-not resuscitate status [12].

### Measurements

We extracted the baseline characteristics of the population from the ANDROMEDA-SHOCK database: age, sex, weight, acute physiology and chronic health evaluation (APACHE II) score, sequential organ failure assessment (SOFA) score, Charlson comorbidity index and source of infection. We also reported the need for mechanical ventilation and renal replacement therapy (RRT), the number of vasopressors-free days as well as intensive care unit (ICU) and hospital lengths of stay, and in-hospital mortality.

The initial dose of NE and other hemodynamic variables were recorded at the randomization point, within 4 h of septic shock diagnosis. NE dose, DAP and HR were recorded simultaneously at this time point.

We calculated VNERi as the DAP/(NE dose x HR) ratio after NE was started. The DAP was expressed in mmHg, HR in beats/min and NE dose in µg/kg/min (using the bitartrate formulation of NE).

### Outcomes

The primary endpoint was to compare the association between DAP alone, DAP/HR, MAP/NE dose and VNERi with in-hospital mortality. Secondary endpoints included the comparisons between the associations of DAP alone, DAP/HR, MAP/NE dose and VNERi with the number of vasopressor-free days and with the number of RRT-free days recorded up to day 28 (D28).

### Statistics

To describe baseline characteristics of the ANDROMEDA-SHOCK population, categorical variables were expressed as number with percentage (%) and continuous variables as mean with standard deviation (SD), or median with interquartile range [IQR]. Given the low proportion of missing data, no missing data imputation method was used.

Firstly, a univariate regression was constructed to assess and to compare the associations of DAP alone, DAP/HR and VNERi with the in-hospital mortality at the randomization time-point. We also assessed the association of MAP/(NE dose) (already described as a vasopressor responsiveness index) [14] and compared it to VNERi.

Secondly, we set up a multivariate model to evaluate the effect of VNERi on in-hospital mortality, using a logistic regression analysis. The rms package was used for all multivariable analyses [15]. The variables included in the model were: clinical and hemodynamic variables at randomization point, age, weight, APACHE II score, SOFA score, central venous oxygen saturation (ScvO_2_), carbon dioxide pressure difference between central venous blood and arterial blood (PCO_2_ gap), volume of resuscitation fluid before randomization. The multivariate model was also adjusted for NE dose, HR, systolic arterial pressure (SAP), MAP, MAP/(NE dose), DAP, central venous pressure (CVP), mottling score, capillary refill time (CRT), and plasma lactate level. For all the variables included in the multivariate model, a collinearity test was performed using the variance inflation factor [16].

The variable selection process for the full model involved a stepdown approach with a higher significance level (α = 0.5). Here, variables selection approach was adopted utilizing AIC (Akaike Information Criterion) and BIC (Bayesian Information Criterion) values. The AIC and BIC criteria can be used to estimate the prediction error and therefore the relative quality of statistical models for a data set. The AIC and BIC estimate the quality of each model relative to each of the other models. The variable with minimum BIC and AIC was selected. A difference of 2 units of values of AIC or BIC between two variables was considered as significantly different [17]. The variable fit was evaluated using the validate function, which used bootstrapping resampling validation to estimate bias-corrected indices specific to each variable. The multivariate model was retested for validation using bootstrap on 1,000 re-samples. Thirdly, we assessed the contribution of the most relevant variables retained in the multivariate model.

Regarding the analysis of secondary endpoints (number of vasopressors-free days and number of RRT-free days up to D28), their statistical associations with VNERi were evaluated using the same multivariate model.

To analyse the relationship between in-hospital mortality and VNERi, we used the Youden test to identify optimal cut-points for distinguishing between deceased and surviving patients on both the descending and ascending segments of the curve. Based on these cut-points, we classified patients into three subgroups: low VNERi (subgroup 1), moderate VNERi (subgroup 2), and high VNERi (subgroup 3). Pairwise comparisons between subgroups were made using the Games Howell test, and p-values were adjusted using Holm’s method.

All tests were two-tailed, with p-values considered significant if 0.05. Statistical analyses were performed using R software 4.1.2 (The R Foundation for Statistical Computing, http://www.r-project.org).

## Results

### Patient characteristics at baseline

A total of 424 patients admitted for septic shock were included. The mean age was 63 ± 17 years. Mean APACHE II score at inclusion was 22 ± 10. Median time from septic shock diagnosis to study inclusion was 80 min [IQR: 0-180]. Median DAP and MAP were 52 mmHg [IQR: 45–50] and 66 mmHg [IQR: 60–76] respectively. The median NE dose at randomization was 0.2 µg/kg/min [IQR: 0.1–0.4]. In-hospital mortality was 43%. Additional baseline characteristics are presented in Table 1.

**Table 1:** Characteristics at baseline:

Characteristics	*N* = 424
Age, mean (SD), yrs	66 (17)
Sex, %	53
Weight, mean (SD), kg	70 (17)
APACHE II, mean (SD)	22 (8)
SOFA D_1_, mean (SD)	10 (3)
Charlson comorbidity index, median [IQR]	3 [1–5]
Co-morbidities	
Chronic hypertension, %	42
Heart failure, %	7
Chronic kidney disease, %	5
Diabetes mellitus, %	24
Source of sepsis	
Pneumonia, %	30
Urinary tract infection, %	21
Intra-abdominal infection, %	35
Others, %	14
Initial management data, median [IQR]	
Time from septic shock diagnosis to antibiotics, min	120 [60–120]
Time from septic shock diagnosis to inclusion, min	81 [0-180]
Haemodynamic variables at vasopressor start point	
Norepinephrine dose, median [IQR], µg/kg/min	0.2 [0.1–0.4]
SAP, median [IQR], mmHg	100 [85–113]
DAP, median [IQR], mmHg	52 [45–60]
MAP, median [IQR], mmHg	66 [60–76]
PP, median [IQR], mmHg	45 [35–58]
HR, median [IQR], beats/min	103 [87–120]
Central venous oxygen saturation, median [IQR], n	73 [65–79], 401
PCO_2_ gap, median [IQR], mmHg, n	7 [5–10], 398
Central venous pressure, median [IQR], mmHg, n	9 [6–13], 393
Serum lactate, mean (SD), mmol/l, n	4.5 (3.5), 424
Mottling score, median [IQR]	0 (0–2)
Capillary refill time, median [IQR], sec	5 [3–6]
Pre-inclusion received fluid volume, median [IQ], mL	2000 [1200–2800]
In-hospital mortality, %	43

Abbreviations: APACHE II: Acute physiology and chronic health evaluation, SOFA D_1_ sequential organ failure assessment calculated the first day, SAP: systolic arterial pressure, DAP: diastolic arterial pressure, MAP: mean arterial pressure, PP: pulse pressure, HR: heart rate, PCO_2_ gap: carbon dioxide pressure difference between central venous blood and arterial blood

### Univariate regression for association between variables and in-hospital mortality

We used a univariate logistic regression to examine the association between in-hospital mortality and the following variables: DAP, DAP/HR, MAP/NE dose and VNERi. The analysis was conducted on the entire cohort (243 survivors and 181 non-survivors out of 424 patients).

VNERi demonstrated a significantly better performance in terms of AIC (543) and LR chi-square (41.76), (*p* < 0.0001) in the univariate regression compared to DAP/HR (AIC = 572, LR chi -square = 11.14, *p* = 0.008) and to DAP (AIC = 580 and LR chi -square = 2.69, *p* = 0.101). DAP/HR was significantly more associated with in-hospital mortality than DAP, (Delta AIC = 8, *p* < 0.01). Compared to DAP/HR, VNERi was significantly more associated with in-hospital mortality (Delta AIC = 29, *p* < 0.0001). VNERi demonstrated the best quality of fit, with the lowest AIC and BIC values and the best association with in-hospital mortality probabilities. Notably, VNERi also showed a better quality of fit compared with the MAP/NE dose ratio (delta AIC = 21, *p* < 0.0001) (Table 2).

**Table 2:** Univariate regression: association between variables and in-hospital mortality

Variables	LR Chi- Square	Pr > Chi- Square	AIC	BIC
**MAP/(NE dose)**	18	< 0.0001	564	572
**DAP**	2.69	0.101	580	588
**DAP/HR**	11.14	0.008	572	580
**VNERi**	41.76	< 0.0001	543	555

Abbreviations: AIC: Akaike information criterion, BIC: Bayesian information criterion, DAP: diastolic arterial pressure, HR: heart rate, NE dose: norepinephrine dose, VNERi: vascular norepinephrine responsiveness index

VNERi showed the best quality of fit, with the lowest AIC and BIC values implying the best association with the probability of in-hospital mortality in the cohort

### Multivariate regression model for association between variables and in-hospital mortality

**Fig. 1:**
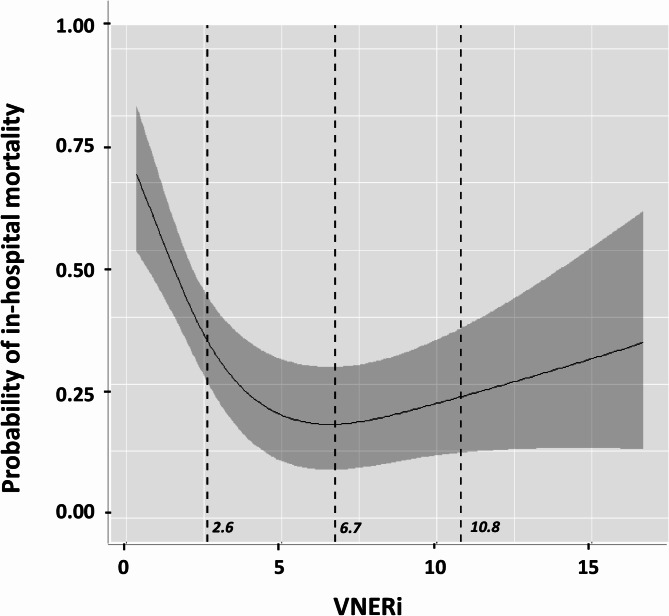
Probability of in-hospital mortality as a function of the VNERi. Abbreviations: APACHE II: Acute physiology and chronic health evaluation, SOFA: sequential organ failure assessment, SAP: systolic arterial pressure, DAP: diastolic arterial pressure, MAP: mean arterial pressure, HR: heart rate, PP: pulse pressure, NE dose: norepinephrine dose. The multivariate regression model revealed an inverted J-shaped relationship between in-hospital mortality and VNERi, with a nadir point at 6.7. The first cut-point defining the descending segment of the inverted J-shaped curve was a VNERi of 2.6. The second cut-point, defining the ascending segment, was a VNERi of 10.8. Multivariate analysis identified a final model with an AIC = 509. Multivariate analysis model was adjusted for the baseline covariates: age, sex, weight, APACHE II and SOFA score, pre-randomisation resuscitation fluid volume in the ANDROMEDA-SHOCK study, NE dose, HR, SAP, MAP, DAP, central venous pressure, mottling score, capillary refill time, plasma lactate level, central venous oxygen saturation, carbon dioxide pressure difference between central venous blood and arterial blood.

Compared to DAP, DAP/HR was significantly more associated with in-hospital mortality (Delta AIC = 10, *p* < 0.01). Compared to DAP/HR and MAP/NE dose, VNERi was more strongly associated with in-hospital mortality (Delta AIC = 7, *p* < 0.05 and Delta AIC = 4, *p* < 0.05, respectively) (Table 3). Figure 1 shows the probability of in-hospital mortality as a function of VNERi. The multivariate analysis identified a final model with an AIC of 509.

**Table 3:** Multivariate regression: association between variables and the in-hospital mortality

Variables	LR Chi- Square	Pr > Chi-Square	AIC	BIC
**MAP/(NE dose)**	86	< 0.0001	513	558
**DAP**	70	< 0.0001	526	562
**DAP/HR**	80	< 0.0001	516	553
**VNERi**	87	< 0.0001	509	546

Abbreviations: AIC: Akaike information criterion, BIC: Bayesian information criterion, DAP: diastolic arterial pressure, HR: heart rate, NE dose: norepinephrine dose, VNERi: vascular norepinephrine responsiveness index

The multivariate analysis model was adjusted for the following baseline covariates: age, sex, weight, APACHE II and SOFA score, pre-randomisation fluid volume in the ANDROMEDA-SHOCK study, NE dose, HR, systolic arterial pressure, mean arterial pressure, DAP, central venous pressure, mottling score, capillary refill time, plasma lactate level, central venous oxygen saturation, carbon dioxide pressure difference between central venous blood and arterial blood

VNERi showed the best quality of fit, with the lowest AIC and BIC values implying the best association with the probability of in-hospital mortality in the cohort

The multivariate regression model revealed an inverted J-shaped relationship between in-hospital mortality and VNERi, with a nadir point at 6.7. The first cut-point defining the descending segment of the inverted J-shaped curve was a VNERi of 2.6. The second cut-point, defining the ascending segment, was a VNERi of 10.8. Based on these cut-points, we identified three distinct patient subgroups. Subgroup 1 (*n* = 214) had a mean VNERi of 1.3 ± 0.9, subgroup 2 (*n* = 182) had a mean VNERi of 5.4 ± 0.3, and subgroup 3 (*n* = 28) had a mean VNERi of 15.8 ± 1.4 (*p* < 0.001 between subgroups). In-hospital mortality was 56 ± 6% in subgroup 1, compared to 28 ± 7% in subgroup 2 (*p* < 0.0001) and 39 ± 19% in subgroup 3 (*p* < 0.0001). Subgroup 3 also had significantly higher in-hospital mortality compared to subgroup 2 (*p* < 0.01) (Supplemental Fig. S1). The DAP/HR ratio was significantly lower in subgroup 1 (0.46 ± 0.01) compared to subgroup 2 (0.65 ± 0.05, *p* < 0.0001) and compared to subgroup 3 (0.82 ± 0.15, *p* < 0.0002) (Supplemental Fig. S2). The NE dose was higher in subgroup 1 compared to subgroup 2 (*p* < 0.0001) and subgroup 3 (*p* < 0.0001). Furthermore, the NE dose in subgroup 3 was lower than in subgroup 2 (*p* < 0.0001) (Supplemental Fig. S3).

### Analysis of the weight of covariates in the final multivariate model

**Fig. 2:**
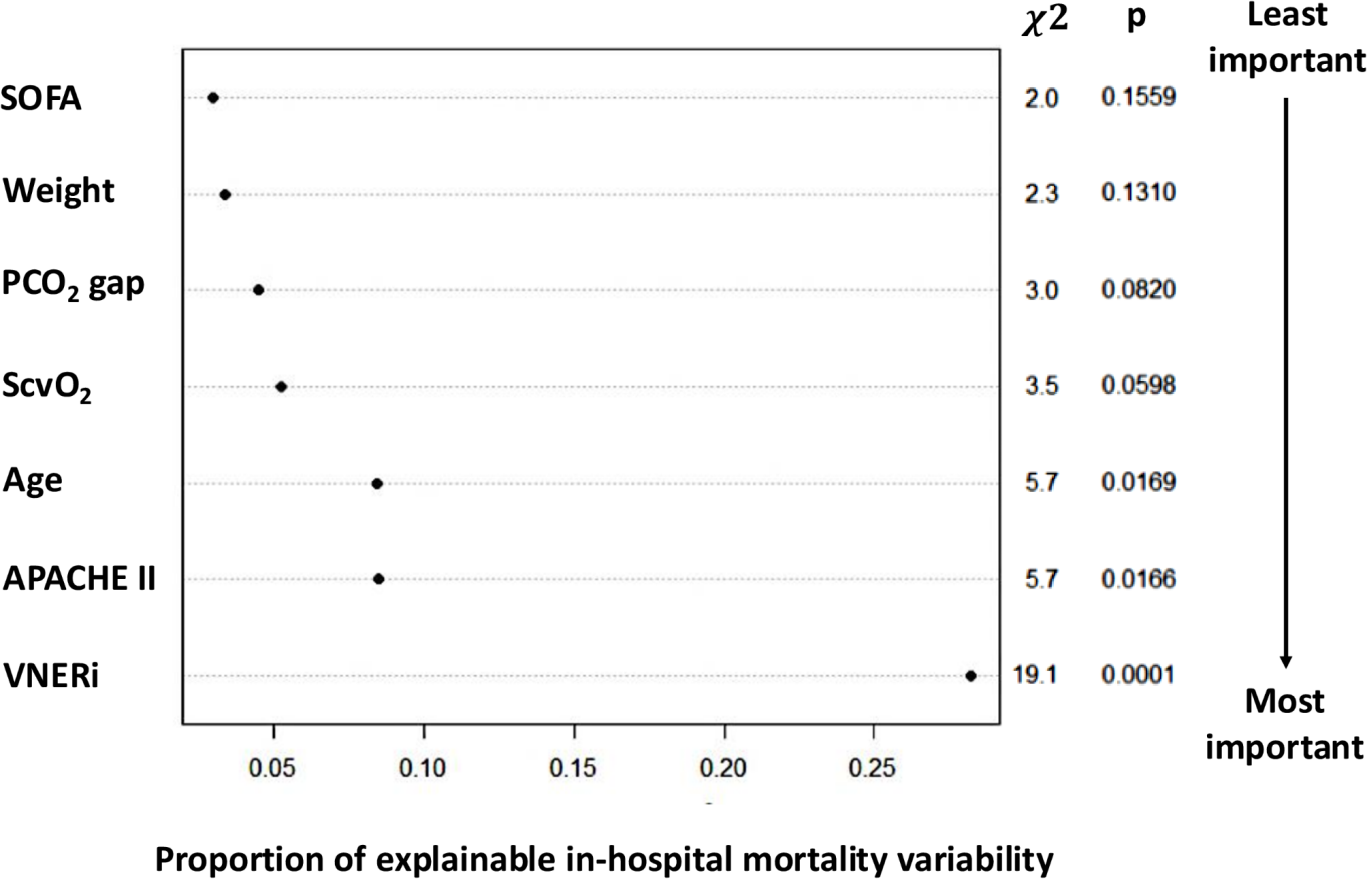
Relative importance of covariates to explain in-hospital mortality variability. Abbreviations: APACHE II: Acute physiology and chronic health evaluation, SOFA: sequential organ failure assessment, DAP: diastolic arterial pressure, HR: heart rate, NE dose: norepinephrine dose, PCO_2_ gap: carbon dioxide pressure difference between central venous blood and arterial blood, ScvO_2_: central venous oxygen saturation

The model identified VNERi as the most important covariate, with a relative importance index (RII) of 46%, independently correlated with in-hospital mortality (OR: 0.33 [IC95%; 0.2–0.56], *p* = 0.0002) after adjustment for prespecified covariates. Other variables associated with in-hospital mortality included age (OR: 1.49 [IC95%; 1.06–2.07], *p* = 0.0197) and APACHE II score (OR: 1.73 [IC95%; 1.12–2.66], *p* = 0.0135), both contributing equally to the model (RII of 14%). ScvO_2_ was associated with in-hospital mortality with an OR of 0.76 [IC95%; 0.58–1.01] (*p* = 0.0588, RII = 8.5%),, PCO_2_ gap with an OR of 1.26 [IC95%; 0.97–1.64] (*p* = 0.0795, RII = 7.3%), the weight with an OR of 0.81 [IC95%; 0.62–1.06] (*p* = 0.1295, RII = 5.6%) and the SOFA score with an OR of 1.32 [IC95%; 0.89–1.97] (*p* = 0.1664, RII = 4.8%) (Table 4; Fig. 2 and Supplemental Fig. S4 and Fig. S5).

**Table 4:** Performance and relative importance of covariates associated with in-hospital mortality using multivariate analysis

Variables	Odds Ratio	Standard error	CI 95%	*p*	RII	Rank
**SOFA**	1.32	0.041	0.89–1.97	0.1664	0.05	7
**Weight**	0.81	0.007	0.62–1.06	0.1295	0.06	6
**PCO**_**2**_ **gap**	1.26	0.027	0.97–1.64	0.0795	0.07	5
**ScvO**_**2**_	0.76	0.010	0.58–1.01	0.0588	0.09	4
**Age**	1.49	0.007	1.07–2.07	0.0197	0.14	3
**APACHE II**	1.73	0.020	1.12–2.66	0.0135	0.14	2
**VNERi**	0.33	0.113	0.20–0.56	0.0002	0.46	1

Abbreviations: SOFA: sequential organ failure assessment, PCO_2_ gap: carbon dioxide pressure difference between central venous blood and arterial blood, ScvO_2_: central venous oxygen saturation, APACHE II: Acute physiology and chronic health evaluation, RII: relative importance index, VNERi: vascular norepinephrine responsiveness index

### Association between VNERi and the need for organ support up to D28

VNERi was significantly associated with the number of vasopressor-free days and the number of RRT-free days up to D28 after adjustment for covariates with a coefficient of 6.052 [CI95%; 3.51–8.59] (*p* < 0.0001), and a coefficient of 6.046 [CI95%; 3.51–8.58] (*p* < 0.0001), respectively. In agreement with the AIC/BIC model of variable selection, compared to DAP alone, DAP/HR and MAP/NE dose, VNERi was more strongly associated with the number of vasopressor-free days and RRT-free days up to D28 (Supplemental Table S2 and S3).

## Discussion

Our study demonstrated that after adjusting for clinical and hemodynamic parameters, VNERi calculated as DAP/(NE dose x HR) at the early phase of septic shock in patients under NE, was more strongly associated with in-hospital mortality than DAP, DAP/HR, or MAP/NE dose. The inflection point of the mortality curve was identified at a VNERi value of 6.7 below which mortality increased significantly. Moreover, VNERi was also more strongly associated with the number of vasopressor-free days and with the number of RRT-free days up to D28 than DAP, DAP/HR or MAP/NE dose.

We hypothesized that VNERi, by adjusting DAP/HR (an index of vasomotor tone) to the NE dose, reflects vascular responsiveness to NE. For this hypothesis to be valid, VNERi needed to demonstrate a stronger association with outcomes than DAP, DAP/HR, and MAP/NE dose. This was confirmed through analyses using AIC and BIC statistical methods, which balance model fit and complexity.

Our aim was not to create a new severity score that perfectly predicts mortality in septic shock. As shown in Fig. 1, the relationship between VNERi and mortality followed an inverted J-shaped curve, suggesting that vascular hyporesponsiveness to NE in the early phase of septic shock is not the sole determinant of mortality.

Further analysis of this inverted J-shaped relationship revealed a mortality rebound in the subgroup of patients (*n* = 28) with VNERi values greater than 10.8 (subgroup 3). Interestingly, the DAP/HR ratio was higher in subgroup 3 compared to subgroup 1, suggesting that vasomotor tone was less reduced in subgroup 3 than in subgroup 1. It is well-established that septic shock is not solely characterized by vasomotor tone depression. Various mechanisms—including hypovolemia, vascular tone depression, and myocardial depression—can interplay in unique combinations that vary between patients. Therefore, it is possible that inadequately corrected hypovolemia and/or cardiac dysfunction played a more significant role than vasomotor tone depression in the early circulatory failure of patients with high VNERi values at inclusion. This could have ultimately contributed to organ dysfunction and a mortality rate of 39% in this subgroup.

Markers of vasomotor tone have been proposed for bedside use. Calculated SVR has been suggested, since some hemodynamic monitors can display its value. However, measuring SVR is challenging as it requires cardiac output data, which is often unavailable in the early phase of septic shock. Moreover, SVR calculation based on the difference between MAP and CVP is flawed due to the vascular waterfall effect [18–20], leading to an overestimation of the true peripheral vascular resistance [19, 20].

A simpler way to assess the vasomotor tone state is by considering DAP. Physiologically, arterial pressure comprises a static component (MAP) and a pulsatile component, pulse pressure (PP). MAP depends on both cardiac output (stroke volume x HR) and peripheral vascular resistance, while PP mainly depends on stroke volume and arterial stiffness [21]. Thus, DAP is determined by MAP and PP in two opposite ways, increasing with MAP and decreasing with PP. As stroke volume is a common factor to MAP and PP, it does not independently influence DAP [5, 6]. Given that HR also influences DAP, it is logical to adjust for HR when interpreting DAP as a marker of vasomotor tone. Accordingly, the diastolic shock index (DSI), calculated as the HR/DAP ratio, has been shown to have a stronger association with mortality than DAP alone [11]. Nevertheless, while DAP/HR is considered a marker of vasomotor tone, it cannot reflect the severity of vasomotor tone dysfunction in terms of responsiveness to NE. For instance, certain factors, such as vasodilating sedative drugs that reduce vasomotor tone, may not impact vascular responsiveness, resulting in low DAP/HR but non-low VNERi values. Conversely, similar DAP/HR values may be observed in two different septic patients receiving different NE doses (e.g., 0.5 µg/kg/min and 0.05 µg/kg/min). These differences in NE dosing result in distinct VNERi values, reflecting varying degrees of vascular hyporesponsiveness to NE.

A previous analysis of the ANDROMEDA-SHOCK database showed that the DSI, the inverse of DAP/HR, was a better prognostic factor than DAP [11]. However, it did not demonstrate that the DSI x NE dose product was superior to DSI alone, likely due to the omission of other covariates. In the present study, we used AIC and BIC to systematicallycompare variablesaccounting for numerous covariates to provide a more robust approach in identifying variable the most associated with in-hospital mortality.

Knowledge of the VNERi value can help guiding therapeutic strategies in case of persistent hypotension under NE. A low VNERi value, as a marker of vascular hyporesponsiveness to NE, might prompt the clinician to implement a multimodal vasopressor therapy strategy (e.g., introduction of non-catecholaminergic agents like vasopressin or corticosteroids) instead of merely increasing NE doses [22, 23]. Increasing NE doses can lead to serious adverse effects including myocardial injury, tachyarrhythmia, mesenteric, and digital ischemia [24], potentially increasing mortality [25]. Furthermore, in cases of very low VNERi values which indicate vascular hyporesponsiveness to NE, further NE dose escalation is likely to be ineffective to achieve the predefined target MAP, resulting in prolonged hypotension. However, we acknowledge that our hypothesis will require further studies to confirm our findings.

Recent literature has proposed other indices to assess vasomotor tone dysfunction. In paediatric refractory septic shock, the ratio of SVR to the vasoactive inotropic score (VIS), termed vascular reactivity index [26, 27], has been associated with mortality. However, it has notable limitations: SVR overestimates true peripheral vascular resistance [19, 20] and requires cardiac output measurements, while VIS includes inotropic drugs with vasodilatory effects, which makes it an imperfect reflection of vasoconstrictor load.

Another index, MAP divided by the equivalent dose of NE, showed an independent association with mortality [14, 28]. However, in our study, the MAP/NE dose ratio was less strongly associated with in-hospital mortality and the numbers of vasopressor-free days and of RRT-free days than VNERi. Additionally, the MAP/NE dose cannot fully capture vasomotor tone responsiveness to NE, as MAP is influenced by cardiac output. During early septic shock, NE administration can increase cardiac output by increasing cardiac preload [29–31] and contractility [32]. Thus, MAP and cardiac output may both rise with a given NE dose, even in cases of vascular hyporesponsiveness to NE. Additionally, calculating NE dose equivalents when other vasopressors are used introduces further challenges, as converting doses between vasopressors is not straightforward [33, 34]. Importantly, in our study, no other vasoconstrictors were used at the time of inclusion, ensuring clarity in VNERi calculations.

Our study has some limitations. First, our proposed index, VNERi lacks a reference method for comparison, as it was not feasible to assess individual vasomotor responses to incremental NE doses. Second, because NE dose is a component of its denominator, VNERi cannot be calculated before initiating NE. However, international guidelines recommend initiating NE early in case of life-threatening hypotension or low DAP [7–10], even before completing fluid administration [35] and using a peripheral venous catheter if necessary [36]. We did not evaluate VNERi beyond the first four hours of septic shock, as the focus was on the early phase. This early phase is critical for decision-making, and bedside VNERi calculation may help guide the selection of optimal vasopressor strategies if the target MAP has not been achieved.

## Conclusions

In this study, VNERi, calculated simply as the DAP/(NE dose × HR) ratio in early septic shock, was more strongly associated with in-hospital mortality and numbers of vasopressor-free days and RRT-free days up to D28 than DAP, DAP/HR, or MAP/NE dose. These findings provide indirect support for the physiological hypothesis that VNERi reflects vasomotor tone responsiveness to NE. However, further prospective studies are needed to validate its utility in guiding early resuscitation strategies in septic shock.

## Electronic supplementary material

Below is the link to the electronic supplementary material.

Supplementary Material 1

## Data Availability

The datasets used and/or analysed during the current study are available from the corresponding author on reasonable request.

## Abbreviations

AIC: Akaike Information Criterion
APACHE II: Acute physiology and chronic health evaluation
BIC: Bayesian Information Criterion
CRT: Capillary refill time
CVP: Central venous pressure
DAP: Diastolic arterial pressure
HR: Heart rate
ICU: Intensive care unit
IQR: Interquartile range
MAP: Mean arterial pressure
NE: Norepinephrine
PCO_2_ gap: Carbon dioxide pressure difference between central venous blood and arterial blood
SAP: Systolic arterial pressure
ScvO_2_: Central venous oxygen saturation
SD: Standard deviation
SOFA: Sequential organ failure assessment
VNERi: Index of vascular responsiveness to norepinephrine
