# Supplementary material for: Ability of diastolic arterial pressure to better characterize the severity of septic shock when adjusted for heart rate and norepinephrine dose
Source: Ann Intensive Care. 2025 Mar 26;15:43. doi: 10.1186/s13613-025-01454-y (PMC11937472; doi:10.1186/s13613-025-01454-y)
Supplement: Supplementary file 1 — Supplementary Material 1 [file 13613_2025_1454_MOESM1_ESM.docx]

**AOIC-D-24-00816 Revision 2 Additional files**

**Ability of diastolic arterial pressure to better characterize the severity of septic shock when adjusted for heart rate and norepinephrine dose**

Antoine GOURY^1,2^, MD; Zoubir DJERADA^2^, MD, PhD; Glenn HERNANDEZ^3^, MD, PhD; Eduardo KATTAN^3^, MD, PhD; Romain GRIFFON^1^, Gustavo OSPINA-TASCON^4,5^, MD, PhD; Jan BAKKER^6,7^, MD, PhD; Jean-Louis TEBOUL^2,8^, MD, PhD; Olfa HAMZAOUI^1,2^, MD, PhD.

^1^ CHU Reims, Unité de Médecine Intensive et Réanimation Polyvalente, F-51100 Reims, France

^2^ Université de Reims Champagne-Ardenne, Unité HERVI "Hémostase et Remodelage Vasculaire Post-Ischémie" - EA 3801, F-51100 Reims, France

^3^ Departamento de Medicina Intensiva, Facultad de Medicina, Pontificia Universidad Católica de Chile, Santiago, Chile.

^4^ Department of Intensive Care Medicine, Fundación Valle Del Lili, Cali, Colombia.

^5^ Translational Research Laboratory in Critical Care Medicine (Translab-CCM), Universidad Icesi, Cali, Colombia

^6^ Department Intensive Care Adults, Erasmus MC University Hospital Rotterdam, Netherlands

^7^ Division of Pulmonology, Critical Care and Sleep Medicine, Columbia University Irving Medican Center, New York, USA

^8^ Faculté de Médecine Paris-Saclay, Université Paris-Saclay, Le Kremlin-Bicêtre, France

***Corresponding author:***

Antoine GOURY, M.D.

Intensive care Department, Reims University Hospitals, Reims, France

**5 Rue du general Koenig, 51100 Reims, France**

**Tel : (+33) 6 80 63 36 [21](C:\\Users\\FiercePC\\Downloads\\21  Orcid: https:\\orcid.org\\0000-00)**

[Orcid: https://orcid.org/0000-00](C:\\Users\\FiercePC\\Downloads\\21  Orcid: https:\\orcid.org\\0000-00)01-7888 8570

**e-mail :** [agoury@chu-reims.fr](mailto:agoury@chu-reims.fr)

**Legends to figures**

**Supplemental Fig. S1:** Comparison of the probability of in-hospital mortality between subgroup of patients according their VNERi.

Abbreviation: VNERi: vascular norepinephrine responsiveness index.

Subgroup 1 (n = 214) had a mean VNERi of 1.3 ± 0.9, subgroup 2 (n = 182) had a mean VNERi of 5.4 ± 0.3, and subgroup 3 (n = 28) had a mean VNERi of 15.8 ± 1.4.

Pairwise comparisons between subgroups were made using the Games Howell test, and p-values were adjusted using Holm's method.

**Supplemental Fig. S2:** Comparison of the DAP/HR ratio between subgroup of patients according their VNERi.

Abbreviations: DAP: diastolic arterial pressure, HR: heart rate.

Subgroup 1 (n = 214) had a mean VNERi of 1.3 ± 0.9, subgroup 2 (n = 182) had a mean VNERi of 5.4 ± 0.3, and subgroup 3 (n = 28) had a mean VNERi of 15.8 ± 1.4.

Pairwise comparisons between subgroups were made using the Games Howell test, and p-values were adjusted using Holm's method.

**Supplemental Fig. S3:** Comparison of the NE dose between subgroup of patients according their VNERi.

Abbreviation: NE dose: norepinephrine dose.

Subgroup 1 (n = 214) had a mean VNERi of 1.3 ± 0.9, subgroup 2 (n = 182) had a mean VNERi of 5.4 ± 0.3, and subgroup 3 (n = 28) had a mean VNERi of 15.8 ± 1.4.

Pairwise comparisons between subgroups were made using the Games Howell test, and p-values were adjusted using Holm's method.

**Supplemental Fig. S4:** Relation between covariates and log odds of in-hospital mortality.

Abbreviations: APACHE II: Acute physiology and chronic health evaluation, SOFA: sequential organ failure assessment, DAP: diastolic arterial pressure, HR: heart rate, NE dose: norepinephrine dose, PCO_2_ gap: carbon dioxide pressure difference between central venous blood and arterial blood, ScvO_2_: central venous oxygen saturation.

**Supplemental Fig. S5**: Forest plot of the adjusted Odds Ratio for the association between variables and the in-hospital mortality.

|  | Item No. | Recommendation | Page  No. |
| --- | --- | --- | --- |
| **Title and abstract** | 1 | (*a*) Indicate the study’s design with a commonly used term in the title or the abstract | 1 |
|  |  | (*b*) Provide in the abstract an informative and balanced summary of what was done and what was found | 2,3 |
| Introduction | | | |
| Background/rationale | 2 | Explain the scientific background and rationale for the investigation being reported | 5 |
| Objectives | 3 | State specific objectives, including any prespecified hypotheses | 6 |
| Methods | | | |
| Study design | 4 | Present key elements of study design early in the paper | 6 |
| Setting | 5 | Describe the setting, locations, and relevant dates, including periods of recruitment, exposure, follow-up, and data collection | 6 |
| Participants | 6 | Give the eligibility criteria, and the sources and methods of selection of participants. Describe methods of follow-up | 7 |
| Variables | 7 | Clearly define all outcomes, exposures, predictors, potential confounders, and effect modifiers. Give diagnostic criteria, if applicable | 7 |
| Data sources/ measurement | 8* | For each variable of interest, give sources of data and details of methods of assessment (measurement). Describe comparability of assessment methods if there is more than one group | 7 |
| Bias | 9 | Describe any efforts to address potential sources of bias | NA |
| Study size | 10 | Explain how the study size was arrived at | NA |

**Supplemental Table S1:** STROBE checklist.

| Quantitative variables | 11 | Explain how quantitative variables were handled in the analyses. If applicable, describe which groupings were chosen and why | NA |
| --- | --- | --- | --- |
| Statistical methods | 12 | (*a*) Describe all statistical methods, including those used to control for confounding | 8 |
|  |  | (*b*) Describe any methods used to examine subgroups and interactions | 9 |
|  |  | © Explain how missing data were addressed | NA |
|  |  | (*d*) *Cohort study*—If applicable, explain how loss to follow-up was addressed | NA |
|  |  | (*e*) Describe any sensitivity analyses | NA |
| Participants | 13* | (a) Report numbers of individuals at each stage of study—eg numbers potentially eligible, examined for eligibility, confirmed eligible, included in the study, completing follow-up, and analysed | NA |
|  |  | (b) Give reasons for non-participation at each stage | NA |
|  |  | (c) Consider use of a flow diagram | NA |
| Descriptive data | 14* | (a) Give characteristics of study participants (eg demographic, clinical, social) and information on exposures and potential confounders | 7,8 |
|  |  | (b) Indicate number of participants with missing data for each variable of interest |  |
|  |  | (c) *Cohort study*—Summarise follow-up time (eg, average and total amount) | NA |
| Outcome data | 15* | *Cohort study*—Report numbers of outcome events or summary measures over time | NA |

**Results**

| Main results |  | (*a*) Give unadjusted estimates and, if applicable, confounder-adjusted estimates and their precision (eg, 95% confidence interval). Make clear which confounders were adjusted for and why they were included | 10,11,12 |
| --- | --- | --- | --- |
| Other analyses | 17 | (*b*) Report category boundaries when continuous variables were categorized | NA |
| **Discussion** |  |  |  |
| Key results | 18 | (*c*) If relevant, consider translating estimates of relative risk into absolute risk for a meaningful time period | 12 |
| Limitations | 19 | Discuss limitations of the study, taking into account sources of potential bias or imprecision. Discuss both direction and magnitude of any potential bias | 15,16 |
| Interpretation | 20 | Give a cautious overall interpretation of results considering objectives, limitations, multiplicity of analyses, results from similar studies, and other relevant evidence | 16 |
| Generalisability | 21 | Discuss generalizability (external validity) of the study results | NA |
| Other information | |  |  |
| Funding | 22 | Give the source of funding and the role of the funders for the present study and, if applicable, for the original study on which the present article is based | 18 |

**Supplemental Table S2:** Multivariate regression: association between variables and the number of vasopressor-free days up to day 28.

| **Variables** | **LR Chi-**  **Square** | **Pr >**  **Chi-Square** | **AIC** | **BIC** |
| --- | --- | --- | --- | --- |
| **MAP/(NE dose)** | 96 | <0.0001 | 3247 | 3295 |
| **DAP** | 80 | <0.0001 | 3262 | 3310 |
| **DAP/HR** | 86 | <0.0001 | 3257 | 3306 |
| **VNERi** | 101 | <0.0001 | 3242 | 3291 |

Abbreviations: AIC: Akaike information criterion, BIC: Bayesian information criterion, DAP: diastolic arterial pressure, HR: heart rate, NE dose: norepinephrine dose, VNERi : vascular norepinephrine responsiveness index.

The multivariate analysis model was adjusted for the following baseline covariates: age, sex, weight, APACHE II and SOFA score, pre-randomisation fluid volume in the ANDROMEDA-SHOCK study, NE dose, HR, systolic arterial pressure, mean arterial pressure, DAP, central venous pressure, mottling score, capillary refill time, plasma lactate level, central venous oxygen saturation, carbon dioxide pressure difference between central venous blood and arterial blood.

VNERi showed the best quality of fit, with the lowest AIC and BIC values implying the best association with the number of vasopressor free days up to day 28.

**Supplemental Table S3:** Multivariate regression: association between variables and the number of renal replacement therapy-free days up to day 28.

| **Variables** | **LR Chi-**  **Square** | **Pr >**  **Chi-Square** | **AIC** | **BIC** |
| --- | --- | --- | --- | --- |
| **MAP/(NE dose)** | 97 | <0.0001 | 3245 | 3293 |
| **DAP** | 81 | <0.0001 | 3261 | 3309 |
| **DAP/HR** | 85 | <0.0001 | 3257 | 3305 |
| **VNERi** | 101 | <0.0001 | 3240 | 3289 |

Abbreviations: AIC: Akaike information criterion, BIC: Bayesian information criterion, DAP: diastolic arterial pressure, HR: heart rate, NE dose: norepinephrine dose, VNERi : vascular norepinephrine responsiveness index.

The multivariate analysis model was adjusted for the following baseline covariates: age, sex, weight, APACHE II and SOFA score, pre-randomisation fluid volume in the ANDROMEDA-SHOCK study, NE dose, HR, systolic arterial pressure, mean arterial pressure, DAP, central venous pressure, mottling score, capillary refill time, plasma lactate level, central venous oxygen saturation, carbon dioxide pressure difference between central venous blood and arterial blood.

VNERi showed the best quality of fit, with the lowest AIC and BIC values implying the best association with the number of renal replacement therapy-free days up to day 28.

**Supplemental Fig. S1.**

**Supplemental Fig. S2.**

**Supplemental Fig. S3.**

**Supplemental Fig. S4.**

**Supplemental Fig. S5.**
